# Autoimmune Dry Eye without Significant Ocular Surface Co-Morbidities and Mental Health

**DOI:** 10.3390/vision4040043

**Published:** 2020-10-10

**Authors:** Zahra Ashena, Radhika Dashputra, Mayank A. Nanavaty

**Affiliations:** 1 Sussex Eye Hospital, Brighton & Sussex University Hospitals NHS Trust, Eastern Road, Brighton BN2 5BF, UK; zashena@nhs.net (Z.A.); radhika.dashputra@nhs.net (R.D.); 2 Brighton & Sussex Medical School, University of Sussex, Falmer, Brighton BN1 9PX, UK

**Keywords:** dry eye, autoimmune disease, rheumatoid arthritis, Sjogren’s syndrome

## Abstract

Dry eye symptoms can negatively affect the psychological, physical, and social functioning, which can potentially impair the health-related quality of life. This review evaluated the association between autoimmune related dry eye in the absence of significant ocular surface co-morbidities and mental health. This review found a significantly higher prevalence of mental health disorders (such as depression and anxiety) in systemic lupus erythematous, rheumatoid arthritis, systemic sclerosis, Behcet’s disease, and primary Sjogren’s syndrome patients when compared to the general population. Moreover, patients with depression and anxiety interpret ocular sensations differently than healthy controls and the perception of dry eye symptoms can be influenced by their mood. Somatization is common in depression, and this could influence the perception of ocular discomfort. Anti-depressants and anxiolytics with their potential side effects on the tear film status may also contribute or aggravate the dry eye symptoms in these patients. Although ophthalmologists manage the dry eye disease, as per standardized algorithms, they should be mindful of different ocular sensation interpretation and coexistent mental health issues in a large number of this patient group and initiate a multidisciplinary management plan in certain cases. While rheumatologists look after their autoimmune condition, it may be worth liaising with GP and/or psychiatrist colleagues in order to address their neuropathic type pain and mental health co-morbidities.

## 1. Introduction

Keratoconjunctivitis sicca (KCS) is one of the dry eye syndromes characterized by a deficiency of the aqueous layer of the tear film [1]. KCS can co-exist with several autoimmune disorders with or without the diagnostic criteria of Sjogren’s syndrome [2,3]. When the diagnostic criteria for Sjogren’s Syndrome is fulfilled the condition can be primary Sjogren’s syndrome (pSS), a systemic autoimmune disorder that is characterized by focal lymphocytic infiltration of the exocrine glands with a classic triad of mucosal dryness, positive biopsy, and serologic evidence of autoantibodies or secondary Sjogren’s syndrome (sSS) with glandular features as a late complication in patients with other autoimmune disorders, such as rheumatoid arthritis (RA), systemic lupus erythematosus (SLE), and scleroderma [4]. The diagnostic criteria for primary and secondary Sjogren’s syndrome have been revised and standardised [5]; therefore, the presence of sicca symptoms in patients with connective tissue diseases should not routinely be mistaken for Sjogren’s syndrome. “The principal pathologic mechanism of Sjogren syndrome dry eye is lymphocytic infiltration of the lacrimal glands which leads to an aqueous deficient dry eye [6]. Other proposed mechanisms include meibomian gland dysfunction secondary to immune cells infiltration [7], the loss of conjunctival goblet cells [8], and pro-inflammatory cytokines that are secreted in the tear fluid [9].

Secondary Sjogren’s syndrome has a prevalence of 4% to 50% by diagnostic criteria, disease duration, and geographic region in RA [10,11,12,13]. A study on incidence of dry eye in RA patients reported 10% of patients with Sjogren’s syndrome criteria and as high as 90% of the non-Sjogren patients with dry eye [2]. It was proposed that the cause of dry eye in RA patients might be a result of local pathology affecting the tear fluid, conjunctiva, or cornea, and not always related to sSS.

As many as one-third of the SLE patients are reported to suffer from dry eye syndrome (KCS) [9,14]. Similar to RA, dry eye syndrome in SLE patients was believed to be caused by secondary Sjogren’s syndrome [15]. However, there is increasing evidence for possible independency of dry eye syndrome and sSS in patients that suffer from autoimmune diseases [3]. Likewise, dry eye disease has been identified to occur in 37–79% of patients with systemic sclerosis [16,17].

This review aims to assess the associations between autoimmune related dry eye in the absence of significant ocular surface co-morbidities and mental health in order to increase the awareness among ophthalmologists with regards to the management of refractory cases.

## 2. Association between Autoimmune Dry Eye and Mental Health

Dry eye symptoms can negatively affect psychological, physical, and social functioning [18], which can potentially impair the health-related quality of life [19].

Studies evaluating the association between autoimmune related dry eye and mental health mainly focus on patients with Sjogren’s syndrome as the main representative of this group compared to the general population with no underlying systemic diseases. A significantly higher prevalence of mental health disorders has been consistently demonstrated in pSS patients as compared to the general population. Wan et al. conducted a systematic review on association of dry eye disease with depression and anxiety. Of the 22 studies that were included in their review, 14 were focused on pSS patients. Studies relied on an assorted number of self-administered psychiatric questionnaires in order to affirm the presence and severity of depression and anxiety. Subgroup analysis in patients with pSS showed a higher prevalence of depression (OR = 4.25) and anxiety (OR = 2.67) when compared to matched controls. They reported that prevalence and severity of depression was higher in patients with Sjogren syndrome as compared to healthy individuals with non-Sjogren dry eye [20]. A later study in Chinese population found 33.8% of pSS patients had anxiety and 36.9% suffered from depression [21].

Another study reporting the incidence and structure of anxiety-depressive spectrum disorders in patients with various rheumatic diseases included 180 patients with a reliable diagnosis of SLE, 128 with RA, 110 with systemic sclerosis, 115 with Behcet’s disease, and 80 with primary Sjogren’s syndrome. They used a semi-structured interview in accordance with the ICD-10/DSM-IV in addition to self-administered questionnaires for the diagnosis of mental health disorders. In accordance with the ICD-10/DSM-IV, depressive disorders were identified in 63% of patients overall, including 73% of patients with RA, 50% of patients with SLE, and 49% of pSS. Anxiety disorders were reported in 61.5% of patients overall, including 25% of patients with pSS, 24.5% of patients with SLE and 23% of patients with RA [22]. This highlights the high prevalence of psychological disorders in autoimmune diseases.

While the studies mentioned so far were cross-sectional or case control studies, a retrospective cohort study based on population-based claim data while using Taiwan’s National Health Insurance Research Database investigated the risk of five common psychiatrist-diagnosed disorders in patients with pSS. Three cohorts were assembled—688 patients with newly diagnosed pSS, 3440 patients without pSS or any other underlying autoimmune disease, and 1302 newly diagnosed patients with RA. They found significantly increased incidence of depressive disorder, anxiety disorder, and sleep disorder in patients with pSS when compared to the other two groups. In particular, women with primary Sjogren’s syndrome exhibited a significantly higher risk of developing depressive disorder, anxiety disorder, and sleep disorder. The peak age group of developing depressive disorder was 65–80 years old [23].

It was proposed that the hampered ability to cry in patients with Sjogren’s syndrome may affect their ways of dealing with emotions. A study examining the differences in emotion processing and regulation between people with and without Sjogren’s syndrome found that emotional processing in patients with Sjogren syndrome does not significantly deviate from the normal. However, they found one exception. A relatively large number of patients were alexithymic, i.e., had difficulty identifying and describing feelings [24]. In addition to the irritating ocular symptoms, dry eye in Sjogren syndrome could negatively impact the visual performance and perception of visual function, which may induce and further aggravate the depressive and anxiety symptoms [25]. In addition to the above evidence on the role of dry eye on development of anxiety and depression, there are some data that suggest the amplification of dry eye symptoms in patients with anxiety and depression.

Galor et al. postulated that patients with depression and anxiety may suffer from central sensitization, which affects pain perception and pain related behaviour [26]. Patients with depression and anxiety interpret ocular sensations differently than healthy controls and the perception of dry eye symptoms can be influenced by their mood [27]. Somatization is common in depression, and this could influence the perception of ocular discomfort [28]. Previous studies reported that depression is more closely correlated with dry eye symptoms, but not to dry eye signs, supporting the hypothesis that somatization contributes to the overall dry eye symptoms in depressive patients [26,29].

Another factor contributing to the development of dry eye with concurrent mental health issues are anti-depressants and anxiolytics with their potential side effects on the tear film status [30]. The anti-cholinergic effect of these drugs may contribute to dry eye symptoms due to decreased tear secretion [31]. The number of studies that assess the ocular surface in patients on psychiatric medications is very limited. A study comparing the dry eye in bipolar patients on mood stabilizers (Lithium or Sodium valproate) versus patients on no treatment found decreased tear film breakup time (TBUT) in those on treatment, thus contributing to dry eye [32].

SSRIs (selective serotonin reuptake inhibitor) and SNRIs (serotonin-norepinephrine reuptake inhibitor), which are commonly prescribed for psychiatric diseases due to a relatively low side effect profile, are also associated with an increased risk for dry eye disease. It was demonstrated that the Schirmer test values without topical anaesthetic were significantly lower in patients on SSRI and SNRI when compared to the control group [33].

Overall, the association between autoimmune dry eye and mental health conditions is well documented. However, understanding a cause and effect relationship and the temporal association warrants further prospective investigations.

## 3. Management of Immune Related Dry Eye

These patients suffer from aqueous deficiency and unstable tear film that may be complicated with exaggerated ocular pain and concurrent psychological disorders, as mentioned above. Therefore, the management should address all of these domains. While ophthalmologists manage the dry eye disease, they should be mindful of different ocular sensation interpretation and coexistent mental health issues in a large number of this patient group and initiate a multidisciplinary management plan in certain cases.

## 4. Management of Dry Eye

Management plans are based on algorithms according to the disease severity. However, it may be advisable to treat all concomitant pathologies rather than sticking to an algorithm due to possible coexistence of other ocular comorbidities. A comprehensive examination should be performed to identify the contributing factors and management plan is formulated based on the severity of symptoms, tear film function and stability, ocular surface inflammation, lid margin disease, eyelid malposition, and underlying systemic diseases.

Lifestyle modification: avoid using visual display units for prolonged hours, avoid antihistamines and anticholinergics, avoid smoking, carry out eyelid hygiene, and follow a healthy diet [34].

Topical tear substitutes and lubrication: the first line of treatment in Sjogren has been “volume replacement” and lubrication, which are found to remarkably address symptoms and signs of the disease [35].

Topical steroid: topical steroids as short-term “pulse” treatment have proven safe and effective in improving the TBUT values and ocular surface disease index (OSDI) as well as alleviating the dry eye symptoms [36].

Omega 3 essential fatty acids: these fatty acids block production of IL-1 and TNFα [37] and improve Schirmer test values, TBUT, OSDI, and meibomian gland function in patients with dry eye [38,39,40].

Topical immunosuppressants: efficacy of 0.05% topical Cyclosporine and 0.03% topical Tacrolimus in improving symptoms and signs of moderate to severe dry eye disease have been approved in various RCTs [41,42,43,44].

Punctal plugs: punctal plugs have been proved effective in management of dry eye symptoms and objective signs including TBUT, OSDI and Schirmer measurements [45,46].

Mucolytics: medications like acetylcysteine demonstrated to be effective in management of filamentary keratitis [34].

Autologous serum: this is usually reserved for the most severe cases that have not responded to other treatments. Autologous serum is a non-FDA approved product. In the UK, the approval of NHSBT (NHS Blood and Transplant) is required before prescription. Preparation of this product is expensive and time consuming. Moreover, it needs to be refrigerated and there is high potential risk of contamination with this product [47]. Yet there is inconsistency in the benefits of autologous serum in patients with dry eye disease [48,49,50].

Contact lenses: extended-wear silicone hydrogel soft contact lenses have proved effective in relieving moderate to severe dry eye symptoms in a subgroup of patients with dry eye [51]. The large diameter scleral contact lenses or rigid gas permeable contact lenses are also helpful, as they retain the fluid on the ocular surface [34]. In fact, shielding the sensitized corneal receptors from the triggering environmental stimulus is the main rationale behind the use of scleral lenses. The most effective lens in maintaining the pre-corneal nociceptive barrier is the liquid corneal bandage created by the fluid filled scleral lenses [52].

Secretagogues: medications like oral pilocarpine have cholinergic effect and help with both dry eye and dry mouth. However, their effect on dry mouth is greater than that of dry eye [34] with several systemic side effects [53].

Surgical management of comorbid ocular surface disease: this involves a variety of surgical procedures including punctal diathermy, conjunctivoplasty, repair of entropion/ectropion, and surgical management of lagophthalmos [54].

## 5. Management of Refractory Ocular Pain in Spite of Adequate Dry Eye Treatment

The discrepancy between subjective symptoms and objective signs of dry eye is already reported [26,55]. In fact, symptoms are more linked with factors other than tear film parameters, such as depression, PTSD, and non-ocular pain disorders [26].

The role of neuronal involvement and neurosensory abnormalities in pathophysiology of dry eye is emphasised in DEWS II summary [56]. Two main types of pain are described: nociceptive and neuropathic. Nociceptive pain represents the sensation that is associated with the detection of potentially tissue-damaging noxious stimuli and is protective [57]. Neuropathic pain is defined as “pain caused by a lesion or disease of the somatosensory system” [52].

The features of neuropathic pain include dysesthesia (unpleasant abnormal sensations), spontaneous pain, allodynia (pain response to innoxious stimuli or stimuli that does not normally provoke pain) and hyperalgesia (exaggerated pain to noxious stimuli) [58,59,60]. These patients complain of spontaneous “burning ocular pain”, which can be increased with the wind, light, or heat/cold [58]. This response may be secondary to dysfunction of primary trigeminal afferents (peripheral sensitization) and/or of second and third order neurons (central sensitization) [61]. Peripheral sensitization may be secondary to inflammatory cytokines that are released during tissue damage, while central sensitization results from complex feedback loops within the central nervous system that augment somatosensory pain signalling [62]. Chang et al. assessed the correlation between dry eye symptoms, severity of symptoms, features of neuropathic eye pain, non-ocular pain conditions, and mental health indices in 233 individuals. Their findings were in agreement with previous studies that reported a correlation between dry eye symptoms and anxiety and depression [63]. They noted that dry eye severity was positively associated with neuropathic-like ocular pain complaints and comorbid chronic pain conditions elsewhere in the body. They concluded that, in order to provide the most effective treatment, patients with ocular surface dryness should be considered as a different category from those whose dry eye symptoms are driven by somatosensory dysfunction, such as neuropathy [61].

An additional approach is required in patients with unsatisfactory response to the adequate conventional dry eye treatments and near normal ocular surface condition, given the higher prevalence of psychological disorders in autoimmune diseases [20,21,22,23] and positive correlation between mental health status and severity of dry eye symptoms [26,58,61].

Autologous serum drops and special scleral contact lenses have been tried successfully in sporadic post-LASIK neuralgia cases [64], where neuropathic pain was secondary to peripheral sensitization. Several treatments have been tried in the past in order to manage the neuropathic pain elsewhere in the body, with a central sensitization. Gabapentin and pregabalin, tricyclic antidepressants (TCA), SSRIs, SNRIs, some anti-epileptic medications, and opioids have been tried [65,66,67,68]. The examples include post-herpetic neuralgia and diabetic neuropathic pain. However, there is very limited clinical trials assessing the efficacy of anti-epileptics, anxiolytics, and anti-depressants in neuropathic type ocular pain.

Gabapentin is a molecule that reduces the release of multiple excitatory neurotransmitters and increases the concentration of γ-aminobutyric acid (GABA) in the central nervous system [66]. Pregabalin and Gabapentin have proved to be effective in alleviating the acute post-PRK pain in two cohorts of randomised trials [69,70].

Moreover, Gabapentin has appeared effective in relieving chronic intractable ocular pain based on some reports. Michael et al. reported a case of neuropathic ocular pain in a patient with post-LASIK ectasia, followed by penetrating keratoplasty and glaucoma drainage implant, who responded very well to Gabapentin 300 mg bd after three weeks [71]. Another study reported the efficacy of Gabapentin in relieving intractable neuropathic pain in a glaucomatous blind eye [72]. Galor et al. tried a short course of peri-operative Pregabalin in a double-masked randomised placebo-controlled clinical trial on patients who underwent LASIK given the role of nerve damage in post-LASIK dry eye symptoms [73]. They assessed the dry eye symptoms in their patients 6 months post-operatively and found no significant differences between the groups with regards to dry eye symptoms and ocular pain intensity or neuropathic pain complaints [74].

Ongun et al. conducted a study to evaluate the effect of gabapentin on Sjogren patients with severe dry eye and neuropathic pain. All 72 patients were treated with artificial tears and cyclosporine eye drop, while half of them received additional gabapentin, initiated at 600 mg/day and titrated up to 1800–2400 mg/day as needed for pain relief. They reported significant improvement in OSDI, TBUT and Schirmer test in both groups with a statistically significant difference between groups. The gabapentin group showed meaningful improvement in OSDI, TBUT, and Schirmer test values as compared to the other group. However, they did not report the post-treatment pain score in their cohort of patients. Therefore, the question of gabapentin efficacy in easing the neuropathic eye pain in their Sjogren patients was unanswered. They concluded that the pathophysiology of severe dry eye is not merely limited to the eye and there are central nervous system contributing factors, which are usually not taken into consideration in treatment of severe dry eye [75].

The limitation of our review is that it focuses on immune related dry eye in patients with no significant ocular surface co-morbidities. Therefore, patients with immune related dry eye in the presence of ocular surface disorders, like cicatricial ocular pathologies or limbal stem cell deficiency, are not included in our review, as these pathologies (apart from just the dry eyes) may also have its own contribution towards the causation of any mental health symptoms. The other limitation of this review is that it is a narrative, but not a systematic review. There are a few sporadic case reports in the literature, but striking paucity of RCTs on this subject. Although some studies advocate a multidisciplinary approach or suggest medications for the management of these patients, their recommendations are based on author’s experience or published case reports or they are generalized from available RCTs on management of extra-ocular neuropathic pain. Therefore, it is highly recommended to conduct double-blinded RCTs in future studies to assess the efficacy, posology, and safety of the suggested psychiatric medications in alleviation of neuropathic type ocular pain in patients with autoimmune conditions in the absence of significant ocular surface pathology.

## 6. Summary

It is not uncommon to come across patients with severe dry eye symptoms in the background of Sjogren’s syndrome or other autoimmune diseases without significant ocular surface co-morbidities, who complain of persistent ocular pain despite adequate management of dry eye disease. Their subjective symptoms are out of proportion when compared to the objective signs and they are commonly associated with anxiety, depression, or other mental health problems with reciprocal influence between the dry eye and psychiatric disorders. In fact, their symptoms are more aligned with their mental health status rather than the tear film disfunction. In such cases, frequent exchange of eye drops does not improve the symptoms and unsatisfactory management of these patients is a burden for both the patient and ophthalmologist. We would like to highlight that the presence of below conditions should flag concurrence of neuropathic pain:

- patients with autoimmune conditions who complain of spontaneous burning ocular pain, which may increase with wind, light, or heat/cold [58];
- patients with unsatisfactory response to the appropriate management plan [61];
- discrepancy between symptoms and signs of dry eye [76];
- presence of chronic pain disorders elsewhere in the body [61]; and,
- coexistence of anxiety, depression, PTSD, sleep disorder, or other mental health issues [26].

In such patients, a holistic and multidisciplinary approach would be warranted. While rheumatologists look after their autoimmune condition, it may be worth liaising with GP and/or psychiatrist colleagues in order to address their neuropathic type pain and mental health co-morbidities.
